# STAT5b: A master regulator of key biological pathways

**DOI:** 10.3389/fimmu.2022.1025373

**Published:** 2023-01-23

**Authors:** Madison R. Smith, Lisa R. Forbes Satter, Alexander Vargas-Hernández

**Affiliations:** ^1^ Department of Pediatrics, Division of Immunology, Allergy, and Retrovirology, Baylor College of Medicine, Houston, TX, United States; ^2^ William T. Shearer Texas Children’s Hospital Center for Human Immunobiology, Houston, TX, United States

**Keywords:** STAT5, STAT5b, hematopoiesis, gain of function (GOF), loss of function (LOF)

## Abstract

The Signal Transducer and Activator of Transcription (STAT)-5 proteins are required in immune regulation and homeostasis and play a crucial role in the development and function of several hematopoietic cells. STAT5b activation is involved in the expression of genes that participate in cell development, proliferation, and survival. STAT5a and STAT5b are paralogs and only human mutations in *STAT5B* have been identified leading to immune dysregulation and hematopoietic malignant transformation. The inactivating *STAT5B* mutations cause impaired post-natal growth, recurrent infections and immune dysregulation, whereas gain of function somatic mutations cause dysregulated allergic inflammation. These mutations are rare, and they are associated with a wide spectrum of clinical manifestations which provide a disease model elucidating the biological mechanism of STAT5 by studying the consequences of perturbations in STAT5 activity. Further, the use of Jak inhibitors as therapy for a variety of autoimmune and malignant disorders has increased substantially heading relevant lessons for the consequences of Jak/STAT immunomodulation from the human model. This review summarizes the biology of the STAT5 proteins, human disease associate with molecular defects in STAT5b, and the connection between aberrant activation of STAT5b and the development of certain cancers.

## Introduction

Signal Transducer and Activator of Transcription (STAT) 5 are proteins involved in a variety of critical cellular functions and pathways. STAT5 has two paralogs, STAT5a and STAT5b (1). STAT5 activates in response to a number of cytokines and growth factors. Upon activation, it plays a key role in hematopoiesis, particularly lymphocyte development, proliferation, and survival (2). Mutations in the *STAT5B* gene are linked to impaired protein signaling and function and are associated with stunted growth, autoimmunity and immunodeficiency (3, 4). Hyperactivation of STAT5b is associated with the development of various blood malignancies and tumors and more recently a syndrome of severe allergic inflammation (5, 6). The mechanisms of aberrant STAT5b function are complicated and not all have been fully elucidated. A more robust understanding of how this crucial protein modulates function in the hematopoietic compartment will aid in development of treatment strategies for patients suffering the effects of STAT5b dysfunction.

This review addresses the biology of STAT5 proteins and we discuss STAT5’s role within the context of human disease.

## Biology of STAT proteins

**Figure 1:**
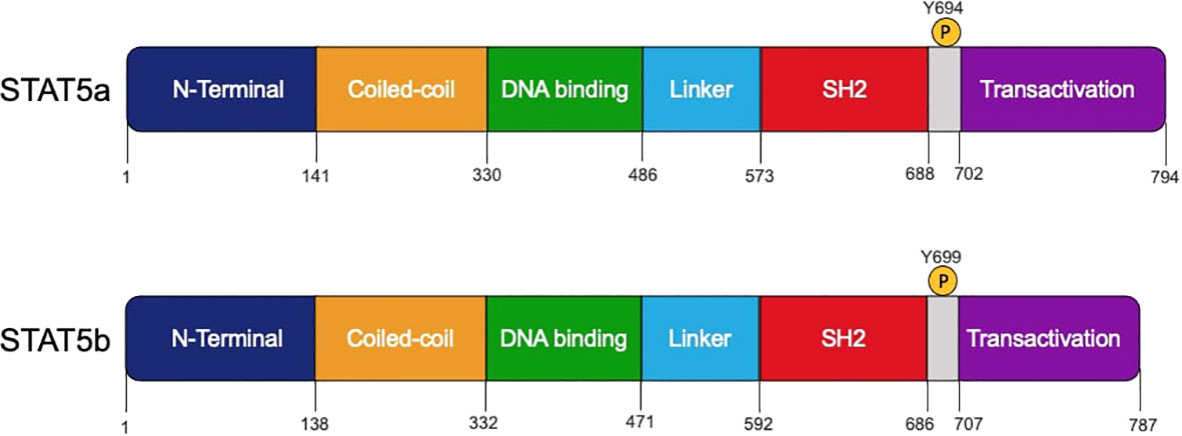
Schematic structures of human STAT5a and STAT5b protein domains. The domains, amino acids residue positions, and the tyrosine 694 and 699 that can be phosphorylated by JAK proteins are shown.

The STAT protein family is named for its dual roles in transducing signal from the cytokine-receptor to the nucleus and activating gene transcription (7). The family includes seven highly related homologous proteins: STAT1, STAT2, STAT3, STAT4, STAT5a and STAT5b (referred to collectively as STAT5), and STAT6 (8–10). Each STAT contains the following conserved domains: an N-terminal region (associated with cooperative DNA binding between dimers) (11, 12), a coiled-coil domain (plays a role during the DNA binding process and provides a site for interactions with other proteins) (10, 13), a DNA binding domain (allows function as transcription factors and targets specific DNA sites) (14), a linker region and SH2 domain (modulates the interaction through phospho-tyrosines) (15, 16), and a C-terminal transactivation domain (17, 18) (**Figure 1**).

### STAT5A/B Gene and Protein

Mammals possess two STAT5 homologs, *STAT5A* and *STAT5B*, and both genes are located on chromosome 17 (19). STAT5a and STAT5b are very similar, with 95% homology between their sequences (1) (**Figure 1**). STAT5a and STAT5b are considered paralogs because they are the protein products of different genes. While they display a high degree of structural similarity, STAT5a differs from STAT5b in several respects. STAT5a contains 12 additional amino acids on the carboxy-terminus and is shortened by five residues on the phosphotyrosyl tail segment (5). The STAT5a and STAT5b DNA binding domains differ by 5 amino acids, which contributes to differences in DNA affinity once they have been activated through phosphorylation and homodimers are formed (20). In addition to these structural differences, STAT5a and STAT5b are phosphorylated at tyrosine at different sites. STAT5a is activated by phosphorylation of a tyrosine found at position 694 while STAT5b is phosphorylated at position 699 (5). Finally, STAT5b is more highly expressed than STAT5a in hematopoietic cell types such as erythrocytes, megakaryocytes, Natural Killer (NK) cells, CD4^+^ and CD8^+^ T cells, and B cells. STAT5a is expressed at higher levels only in CD34^+^ hematopoietic stem cells (20).

**Figure 2:**
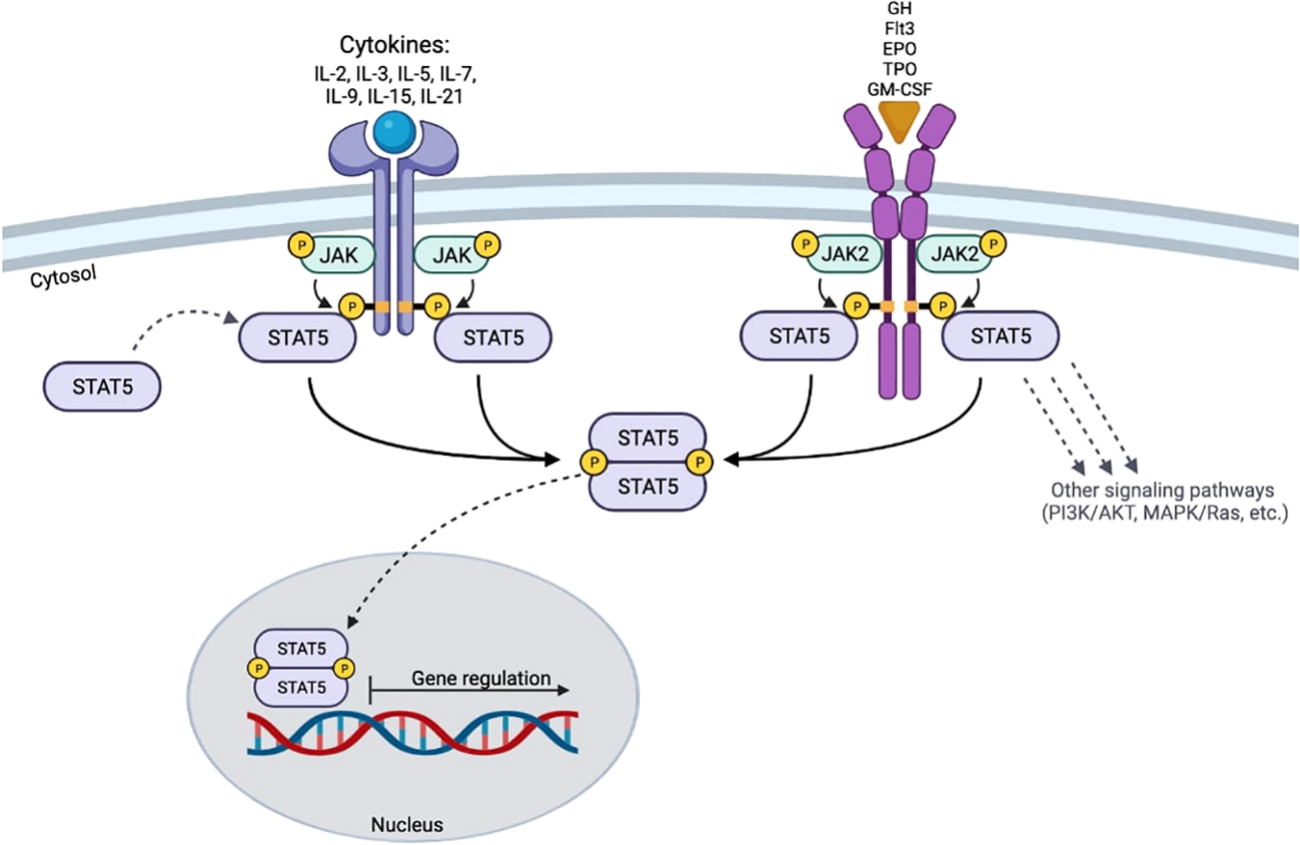
Schematic of STAT5 signaling. The activation of STAT5 requires the binding of cytokines or growth factors to their respective receptors. The cytoplasmic domains of the receptors are trans-phosphorylated by activated JAK proteins. STAT5 proteins then bind to the receptor and become activated by trans-phosphorylation at their specific tyrosine residues. After activation, STAT5 dimerizes and translocates to the nucleus to induce gene transcription.

STAT5 is activated in response to a variety of cytokines (IL-2, -3, -5, -7, -9, -15, -21) (20, 21). Prior to cytokine-receptor interaction, STAT5 proteins are found in a cell’s cytoplasm as inactive antiparallel dimers (10). The canonical JAK-STAT5 signaling pathway is characterized by three sequential tyrosine phosphorylations that occur in response to cytokine and growth factor stimulations (7, 10). After activation, STAT5 proteins form homodimers, heterodimers, or tetramers (7, 22–24). These complexes then translocate to the nucleus, where they induce the transcription of specific genes (2, 25) (**Figure 2**). In the nucleus, the functional dimers are dephosphorylated by phosphatases and signaled for export back to the cytoplasm for further rounds of activation (10). One important canonical signaling pathway for STAT5 includes growth hormone (GH). The interaction between GH and its cell surface receptor (GHR) induces the auto-activation of JAK2, followed by phosphorylation of three tyrosines located in the intracellular domain of the GHR. This sequence of events requires the recruitment and docking of cytosolic STAT5b to the intracellular domain of the GHR (26), activation of STAT5b (tyrosine phosphorylation) by JAK2, dimer formation, and the mobilization of the complex to the nucleus to drive transcriptional programs (3, 27, 28). The GH/GHR system can also activate other STAT proteins such as STAT1, STAT3 (3, 28, 29). STAT5 function can also be initiated by other growth factors reviewed here, including granulocyte-macrophage colony-stimulating factor (GM-CSF), erythropoietin (EPO), thrombopoietin (TPO), and Fms-like tyrosine kinase 3 (Flt3) (20, 21) (**Figure 2**). STAT5 activation downstream of these growth factors is very similar to the pathway initiated by cytokine activation.

STAT5 is also tightly regulated. To begin, the suppressor of cytokine signaling (SOCS) proteins act as negative feedback inhibitor dampening specific cytokine signaling and preventing an excessive response (30). The Cytokine-induced SH2-containing protein (CIS), SOCS1, 2 and 3 are all associated with inhibition of the JAK/STAT signaling pathway (10). While SOCS1 is primarily associated with IFN-γ signaling (31), in the context of IL-2 signaling in T cells, SOCS1 shows a high affinity for regulating cytokines that signal through common gamma chain subunit, such as, IL-2, IL-4, IL-7, and IL-15 (32–34). Conversely, SOCS3 regulates various other signaling pathways by interacting with receptors such as granulocyte colony-stimulating receptor (G-CSFR), leptin receptor, and erythropoietin receptor (EpoR) (35). Different studies demonstrated that during GHR/JAK2/STAT5 signaling, SOCS1 and SOCS3 bind JAK2, leading to degradation of the GHR/JAK2 complex by ubiquitination (30, 36). SOCS2 binds to motifs in the GH receptor through its SH2 domain, inhibiting the STAT5 recruitment (37, 38).

The binding of cytokines to their receptors triggers an intracellular tyrosine phosphorylation cascade, that is also regulated by two other proteins: phosphotyrosines phosphatases (PTPs) and protein inhibitors of activated STATs (PIAS). The PTP protein family includes six members found in both the cytoplasm and nucleus that regulate the intracellular tyrosine phosphorylation cascade. These proteins are constitutively expressed, and therefore are not feedback-inhibitors (30, 39). Finally, the PIAS proteins modulate STAT5 activity in the nucleus by inhibiting the interaction between STAT5-dimers and their DNA promoter regions through SUMOylation of STAT5 (30, 40, 41). STAT5 activation is tightly regulated, importantly so, because the downstream activity of STAT5 has implications on multiple cell types in tissues and the immune system.

## Role of STAT5 proteins during hematopoiesis

**Figure 3:**
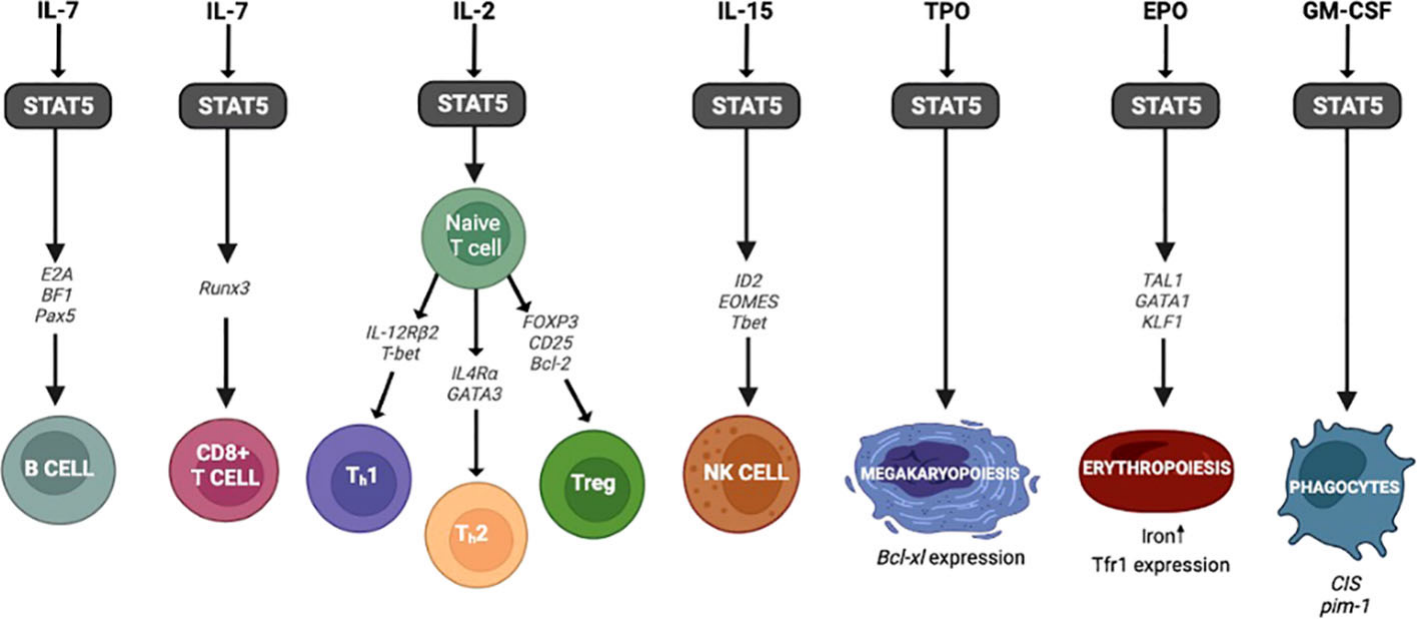
STAT5 and hematopoiesis. The genes and transcription factors involved in hematopoiesis following activation of STAT5.

STAT5 plays a critical role in the development of many hematopoietic lineages, including B cells, T cells, NK cells, erythroid cells, and megakaryocytic cells (**Figure 3**). In regards to hematopoiesis, STAT5b is expressed at higher levels than STAT5a in all of these cell types (20).

STAT5 activation that occurs in conjunction with IL-7R signaling regulates B cell lymphopoiesis by promoting cell survival and regulating the immunoglobulin gene rearrangement in pro-B cells (42). STAT5 also regulates the expression of transcription factors critical during early B cell stages such as E2A, EBF1, and Pax5 (43–46). In *Stat5a/b^null/null^* mice there is a block of B cell development between the pro- and pre- B cell stages (47). However, mice that expressed an N-terminal truncated Stat5a/b protein (*Stat5a/b* ^ΔN/ΔN^ mice*)* showed far less impairment in B cell development than the knockout mice leading to a more mild phenotype. These results suggest that the N-terminal truncated STAT5a/b may partially rescue B cell development, but the mechanism behind this is unclear (47). The activation of STAT5 in response to other cytokines can have distinct effects on B cell differentiation as well. Recently, Pelham and colleagues demonstrated that STAT5b can induce the expression of SOCS3 in response to IL-21 regulating naïve B cell differentiation to memory B cells and plasma cells (48). So, when STAT5b is decreased or absent, as in STAT5b deficient patients, there is a skewing towards terminally differentiated memory and plasma cells compared to wild type (48). Studies such as this demonstrate the ubiquitous function of STAT5b in immune cells and highlight the importance in regulating lymphocyte differentiation (**Figure 3**).

STAT5 plays an important role in the development, differentiation, and survival of T cells (49). To start with data surrounding development, *Stat5a/b^-/-^* mice show severe impairment of lymphocyte development which mimic the findings observed in *Il7r^-/-^* mice (50, 51). The IL-7/STAT5 signaling plays a critical role driving the expression of important transcription factors, such as Runx3, required for CD8^+^ T cell differentiation (52). Moreover, Lin and colleagues demonstrated that STAT5-tetramers are essential for the expansion of antigen-specific activated CD8^+^ T cell (24).

As STAT5 has been shown to have a role in the development and differentiation of CD8^+^ T cells, it is similarly involved with the development of the various CD4^+^ T cell subsets (49). Th1 polarization is induced by IL-12 and expression of Tbet leads to IFN-γ production along with other Th1 cytokines. Liao and colleagues demonstrated that while naïve T cells are unable to respond to IL-12 because they do not express the IL-12 receptor β2 (IL-12Rβ2), IL-2 potentiates Th1 differentiation *via* STAT5 activation by inducing IL-12Rβ2 and Tbet expression (53). Similarly, Th2 polarization requires IL-4 signaling and GATA3 expression (49, 54). Importantly, the activation of STAT5 by IL-2 is first required to induce the expression of IL-4 receptor α expression leading to Th2 differentiation (54–57).

The activation of STAT5 in response to IL-2 is also associated with the development and function of CD4^+^FoxP3^+^ regulatory T cells (Tregs) (58–61). In mature Treg cells, the activation of STAT5b drives and maintains *FOXP3*, *CD25*, and *Bcl-2* gene expression (21, 61, 62). Combined with studies using Il2rb-deficient mice (63, 64), these findings demonstrate that STAT5 acts as a central effector during the development and function of Treg cells. Reports involving patients with STAT5b deficiency show impaired Treg homeostasis and decreased numbers of CD4^+^ and CD8^+^ T cell subsets (61, 65).

NK cells are the third subset of lymphocytes in the peripheral blood whose development is dependent on STAT5 activation (66, 67). IL-15 is required for the generation of fully functional NK cells, and the IL-15 trans-presentation by dendritic cells is vital to prime the terminal NK cell maturation (68–74). NK cell activation through IL-15 receptors induces the recruitment and activation of STAT5 (75, 76). Compared to *Stat5a^−/−^* mice, *Stat5b^−/−^* mice exhibit a profound reduction of NK cell frequencies in peripheral blood with a reduction in their activity in response to IL-2 and IL-15 (77, 78). STAT5 drives the expression of essential transcription factors for NK cell development such as ID2, EOMES, and T-bet, and acts as a regulator of the expression of important effector molecules (perforin, granzymes, and IFN-γ) (79). STAT5 can also form tetramers, and Lin and colleagues showed that STAT5 tetramer-deficient mice (DKI mice) had lower numbers of NK cells and that the absence of tetramers was associated with abnormal maturation and with decreased expression of maturation-associated genes (80). Importantly, owing to STAT5b’s ubiquitous role in the development of multiple lymphocyte cell subsets, these DKI mice presented with decreased and defective proliferation of CD8^+^ T cells along with reduced Treg cell presence and function (80).

Similar to IL-15, STAT5 is the principal downstream effector after IL-2 stimulation. NK cells are activated in response to IL-2, enhancing their cytotoxicity capacity, activating receptor expression and cytokine production (81–83) which is severely impaired in STAT5b deficient patients (25). Taken together, the abnormal NK cell maturation observed in humans mimics the abrogated NK cell terminal maturation observed in *Stat5b* ^-/-^ mice (25, 84).

NK cells are closely related to innate lymphoid cells (ILC). While NK cells are more closely related to group 1 ILC, all groups of ILCs deserve mention in regards to STAT5b biology. The ILC family includes four subsets: ILC1, ILC2, ILC3, and lymphoid tissue inducer cells (85, 86). According to their functional and phenotypic characteristics, they are classified into Group 1 ILCs characterized by T-bet expression and IFN-γ production (87), Group 2 ILCs characterized by IL-5 and IL-13 production along with the expression of the transcription factors GATA3 and RORα, and Group 3 ILCs which are characterized by IL-17 and IL-22 production and are dependent on AHR and RORγt (85). Using a mouse model with decreased numbers of STAT5 alleles, Villarino and colleagues demonstrated that STAT5 is necessary for accumulation of all ILC subsets in lymphoid and nonlymphoid tissues. In this model, mice retaining Stat5a alleles rather than Stat5b alleles seemed to have a larger impact on ILC development. Early developmental precursors were present and consistent with γc and IL-7 deficient mice important because γc cytokines involved with lymphocyte development signal through STAT5b (78). As expected ILC2 progenitors were also present but NK cell and IL-1 progenitors were severely affected; an important finding since it corroborates the notion that STAT5 is a critical multilineage transcription factor (MLTF) that induces ILC development, homeostasis and function (78). These findings show the relevance of STAT5 beyond classic lymphocyte biology since ILCs are typically known for their surveillance and defense in tissues like lungs, skin, and intestines.

STAT5 is a regulator that determines lineage commitment between erythroid and megakaryocytic cell fates (88). Starting with erythroid development, EPO induces the activation of the STAT5/JAK2 pathway (89–93). After activation, STAT5 interacts with the transcription factors TAL1, GATA1, and KLF1 (called “master regulators”), and the resulting complex binds enhancers of specifics genes that induce erythroid differentiation (93–96) (**Figure 3**). In mice, the absence of any one of these transcription factors important to the development of erythroid cells results in severe anemia and death (97–100). Studies with *Stat5a/b* ^-/-^ mice demonstrate that both STAT5a and STAT5b are critical regulators of iron uptake during erythroid development, as the absence of both STAT5 proteins resulted in severe anemia and reduction of Tfr1 expression (transferrin receptor) (101). Finally, chronic anemia was reported in STAT5b deficient patients, further highlighting the role of STAT5b during erythropoiesis (25, 48).

While erythroid cell development is initiated by EPO, megakaryocytic differentiation is activated by TPO *via* JAK/STAT5a/b signaling. TPO signaling activation induces the expression of Bcl-xL and megakaryocytic cell survival (102) (**Figure 3**). Olthof and colleagues reported that the overexpression of constitutively activated Stat5a mutant in CD34^+^ cells in mice induces erythroid differentiation at the expense of megakaryocyte development (88). Furthermore, studies in hematopoietic stem/progenitor cells demonstrated that in the absence of TPO, tyrosine-unphosphorylated STAT5 represses megakaryocytic differentiation by blocking the megakaryocytic transcriptional program (103). The role STAT5 plays in erythroid and megakaryocyte development comes into play when assessing the side effects of suppressing STAT5 through various JAK inhibitor therapies now used for hematopoietic and autoimmune disorders.

Finally, the canonical signaling for the phagocytic growth factor GM-CSF occurs *via* STAT5. While GM-CSF was initially described for its ability to initiate the production of neutrophils and macrophages, it can also participate in the production of eosinophils, erythrocytes, megakaryocytes, and dendritic cells (104–106). Once GM-CSF binds to its receptor (GM-CSFR), the JAK2/STAT5 pathway is initiated leading to downstream gene expression (107). This GM-CSF/STAT5 signaling pathway regulates target genes such as cytokine-inducible SH2-containing protein (*CIS)*, a regulator of STAT proteins (10, 107–109). In addition to its role in phagocytic cell proliferation, GM-CSF/STAT5 signaling is also involved in various functions of alveolar macrophages and the growth of alveolar epithelium in lung tissue (105, 110, 111). These critical alveolar macrophage functions include surfactant and cholesterol clearance (104). Impairment in this signaling pathway can lead to the inability to initiate this protein clearance causing pulmonary alveolar proteinosis (PAP), a known complication in STAT5b deficient patients (112).

## Human defects caused by *STAT5B* mutations

In humans, the identification of deleterious *STAT5B* mutations demonstrates that STAT5a is unable to compensate for the absence of STAT5b, despite their high amino acid similarity, and mutations causing human disease in *STAT5A* have not yet been identified. Pathologic mutations affecting its function and signaling have been described in all domains of *STAT5b* (3, 4). Thus far, different molecular mechanisms of *STAT5B* mutations have been identified. Autosomal recessive loss-of-function (LOF) mutations and heterozygous dominant negative mutations as well as recently, somatic gain-of-function (GOF) mutations, associated with severe allergic inflammation and hematological malignancies, have been reported.

### Loss of Function Mutations

**Figure 4:**
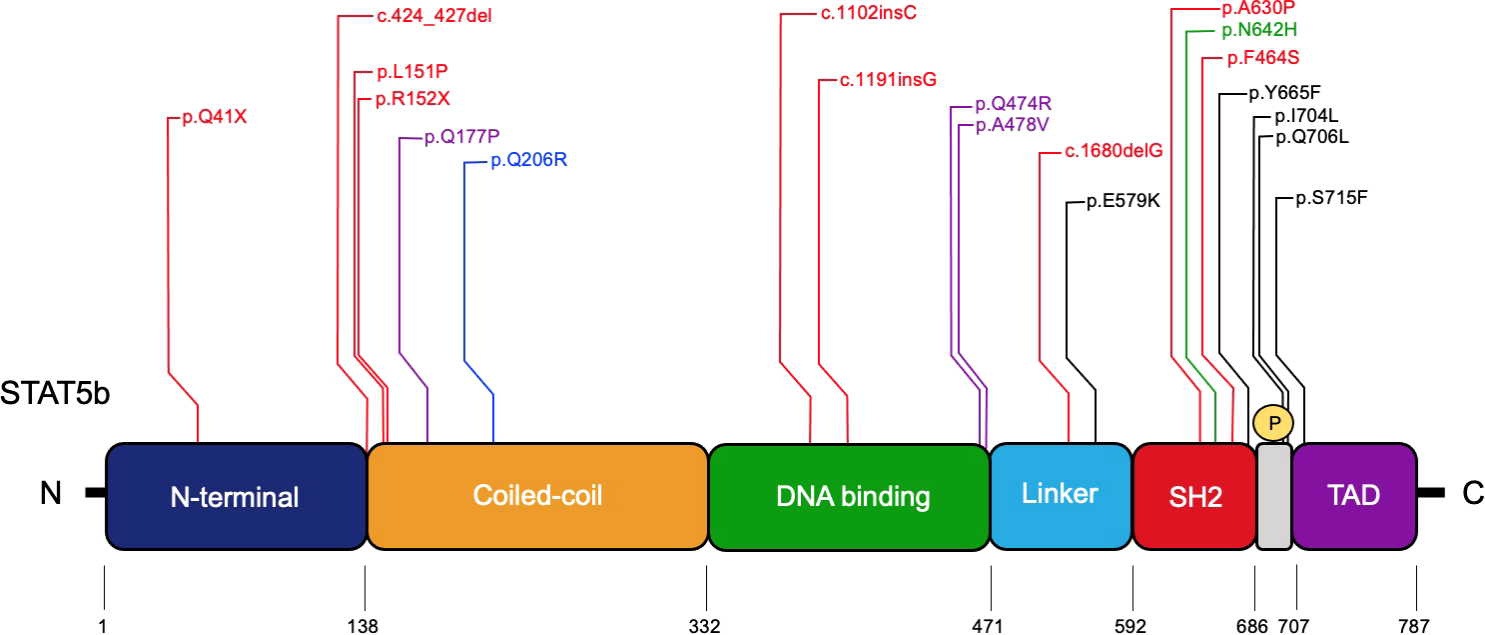
Pathologic *STAT5B* mutations identified in patients. The STAT5b domains and amino acids residue positions are indicated. Red, homozygous LOF mutations; Purple, dominant-negative LOF mutations with growth hormone insensitivity and eczema; Blue, dominant-negative LOF mutation associated with immune dysregulation lacking growth hormone insensitivity; Green, severe allergic somatic GOF mutation; Black, somatic GOF mutations.

Nine homozygous LOF *STAT5B* mutations have seen been reported: three missense mutations (p.A630P, p.F646S, p.L151P) (27, 113, 114); two nonsense mutation (p. Q41X, p.R152X) (48, 115); a deletion of four nucleotides (c.424-427del) (116); two single nucleotide insertions (c.1102insC, c.1191insG) (117, 118); and a single nucleotide deletion (c.1680delG) (119) (**Figure 4**). Nonsense and frameshift mutations predict a truncated STAT5b protein, and the lack of detectable protein in primary cells from these patients suggests that the truncated STAT5b protein is not stably expressed (25, 27, 115, 117, 118). The missense mutation p.A630P located in SH2 domain, was the first mutation described as responsible for STAT5b deficiency (27). Overexpression studies demonstrated that this variant can be expressed, but it cannot be activated by GH or IFN-γ stimulation (27). Furthermore, studies *in silico*, showed that the autosomal recessive p.A630P mutation disrupts the β-sheet core, affecting protein solubility leading to misfolding (120). In addition, two other missense mutations have been described with abnormal functionality and correlate with the clinical STAT5b phenotype (113, 114). Using a reconstitution system, Hwa and colleagues demonstrated that the c.1680delG frameshift mutation was not stably expressed (119).

Loss-of-function mutations are associated with severe forms of pulmonary disease, eczema, combined immunodeficiency, autoimmune disease, bacterial and/or viral infections and post-natal growth delay (**Table 1**). As previously mentioned, STAT5b plays an important role in lymphocyte development, function, and survival; therefore, it would be expected that these patients have recurrent and severe viral infections in addition to bacterial and some reported fungal infections putting these patients at risk for opportunistic infections (21, 61, 84). Autoimmune disease has been reported such as juvenile idiopathic arthritis, interstitial lung disease (ILD), inflammatory bowel disease, Hashimoto’s thyroiditis, and type 1 diabetes (25, 75). The proposed mechanism of autoimmunity in STAT5b deficient patients was reviewed nicely by Kanai et al. where they suggested that STAT5b’s role in driving FOXP3 led to lack of Tregs in these patients. In addition, they suggested that cells are more likely to be susceptible to apoptosis due to lack of STAT5b dependent Bcl2; a protein important for regulation of apoptosis (75).

**Table 1:** Human Disease Manifestations.

Human Disease Manifestations		
Gain of Function	Loss of Function	Dominant Negative
◼ Non-clonal eosinophilia ◼ Urticaria ◼ Dermatitis ◼ Diarrhea ◼ LGL leukemia	◼ Impaired post-natal growth ◼ Low expression of IGF-I, IGFBP-3 and ALS ◼ Elevated prolactin levels ◼ Severe pulmonary disease, eczema, autoimmune disease (juvenile idiopathic arthritis), lung disease, and bacterial and/or viral infections ◼ Low NK cell counts and cytotoxic capabilities ◼ T-lymphopenia (CD4+ and CD8+ T cells) ◼ Reduction of T-regulatory cells ◼ Hypergammaglobulinemia ◼ Chronic Anemia	◼ Impaired post-natal growth ◼ Eczema ◼ Hyper IgE ◼ Growth hormone insensitivity syndrome (GHIS) ◼ Mild immune dysregulation ◼ Low expression of IGF-I, IGFBP-3 and ALS

The clinical manifestations found in patients with GOF, LOF, and dominant negative *STAT5B* variants.

These deleterious loss-of-function *STAT5B* mutations also lead to the disruption of GM-CSF signaling, which is associated with pulmonary alveolar proteinosis (PAP) (104). GM-CSF binds with its heterodimeric receptor, causing activation of JAK2 and initiating multiple pathways. One of these pathways involves STAT5 is important for the development and function of alveolar macrophages (104, 107). Recently, Krone and colleagues described impaired GM-CSF signaling in a patient with STAT5b deficiency (112).

Extra immune manifestations of STAT5b deficiency include growth hormone related abnormalities. Since STAT5b is activated by growth hormone through the GHR, patients with STAT5b deficiency present with normal intrauterine growth but impaired post-natal growth, similar to patients with GHR defects (4). In most cases involving LOF mutations, the endocrine profile reveals a normal basal level of GH, low expression of IGF-1, IGFBP-3 and ALS with elevated prolactin levels, suggesting an altered negative feedback loop for GH signaling (4, 21) (**Table 1**). GH treatment is somewhat challenging because, like GHR deficient patients, there is poor response to endogenous and exogenous GH (27, 117–119). Recently, Bang and colleagues reported a study using recombinant human IGF1 supplementation in patients with GHR defects. They saw a positive gain in linear growth suggesting IGF1 supplementation might be a plausible therapy to promote growth in patients with aberrant growth hormone signaling such as STAT5b deficiency (121).

Heterozygous dominant negative LOF *STAT5B* mutations were recently described in three unrelated patients and are extremely rare (122). These patients only present with impaired post-natal growth and eczema (122). Their characterization provides information about STAT5b as a critical effector for GH signaling and normal human hematopoiesis. Their clinical profile confirmed growth hormone insensitivity syndrome (GHIS), with postnatal growth failure and IGF-I deficiency (122). Two patients developed eczema in the neonatal period and higher levels of IgE (**Table 1**). None experienced severe immune or pulmonary symptoms (122). These missense mutations retain their capacity to become phosphorylated in response to GH and their heterodimer formation capacity is not affected. However, the mutations interfere with the transcriptional activity of the STAT5b wild type. Studies *in vitro* showed that the p.Q177P variant has an abnormal mobilization to the nucleus with an accumulation at the nuclear membrane. The *de novo* mutation is located within an α-helix and studies suggest that it disrupts the secondary structure (122). The p.Q474R and p.A478V variants were characterized with a normal capacity to translocate to nucleus, but their DNA-binding capacity was impaired (122) (**Figure 4**). A new variant, p.Q206R, was discovered in a patient with autoimmunity, chronic lymphadenopathy, and splenomegaly; however, growth hormone insensitivity was not reported (123). This variant, located in the CCD, showed normal expression and similar mobilization to nucleus compared to healthy donors. The authors performed a luciferase reporter assay showing the mutation has a dominant negative effect and correlated this finding with an impaired STAT5 phosphorylation in response to IL-2, IL-7 and IL-15 (123).

### Gain of Function Mutations

Studies on STAT5 GOF mutations shed additional light on pivotal STAT5 functions. The reported N642H variant shows increased STAT5b activity compared to the wild type and promotes upregulation of genes that are required for cell proliferation and survival (124). *In silico* studies show that the *STAT5B^N642H^* variant adopts a conformation that enhances the affinity for self-dimerization and is resistant to dephosphorylation (125). In addition, Ma and colleagues reported two patients with the N642H variant that developed non-clonal eosinophilia, urticaria, dermatitis, and diarrhea (6) (**Table 1**). So far, only somatic STAT5 GOF mutations have been reported, located in the SH2 domain and in the transactivation domain (124, 126–130) (**Figure 4**).

Somatic GOF mutations can also lead to the development of large granular cell (LGL) leukemia in addition to dysregulated activation of STAT5 associated with other types of cancers.

## STAT5 and cancer

LGL leukemia is a chronic proliferation of clonal cytotoxic lymphocytes, frequently complicated with cytopenia and autoimmunity (131). The disease is classified in two subtypes: T cell large granular lymphocyte leukemia (T-LGL) (which represents about 85% of cases) and chronic lymphoproliferative disease of NK cells (NK-LGL) (which represents 10% of cases) (132). Mutations in *STAT5B* are more frequently associated with T-LGL leukemia, more specifically, CD3^+^/CD56^+^ and CD4^+^ subsets of T-LGL leukemia (133). Along with mutations in *STAT5B*, mutated *STAT3* leads to constitutive activation and plays a fundamental role in the pathogenesis of LGL leukemia (133). When evaluating the transcriptome of LGL leukemia patients, many of the genes are regulated by STAT3 (133). There are a couple key differences to note between STAT3 and STAT5b mediated LGL leukemia. One is that CD4^+^ T-LGL leukemia is characterized only by *STAT5B* mutations and not *STAT3* mutations (134). Additionally, *STAT5B* mutations often lead to a more aggressive presentation of the disease (133).

Hyperactivation of STAT5 is also associated with other blood malignancies such as acute lymphoblastic leukemia (ALL), chronic myelogenous leukemia (CML), myeloproliferative neoplasms (MPNs), in addition to human tumors (breast, prostate, liver, etc.), and other cancers (5). Because the dysregulated activation of STAT5 leads to the development of leukemias and myeloproliferative disease, it is important to evaluate the role of STAT5 activators such as tyrosine kinases in these diseases. The TEL-Jak2 fusion protein has been found in patients with pre-B cell and T cell ALL and CML. The fusion of TEL and Jak2 leads to the constitutive activation of its tyrosine activity, followed by constitutive activation of STAT5 (135, 136). Other tyrosine kinase translocation products are also important drivers of cellular transformation and leukemias (137). The relationship between STAT5 and an upstream oncogene plays a critical role in the development of CML. Transformation of cells by BCR-ABL, the oncogene resulting from the fusion of BCR to ABL kinase, is the most common cause of CML. Following translocation and the merging of these genes, the tyrosine kinase activity of ABL increases, over-activating downstream STAT5. The constitutive activation of STAT5 by BCR-ABL then leads to the upregulation of genes associated with CML such as *Bcl-x*, *Mcl-1*, and *D ½*, and this hyperactivation leads to the dysregulated proliferation of cells (136, 138)

Because STAT5 is involved in the development and survival of lymphoid cells, its activation is also correlated with lymphocyte transformation. In a study by Heltemes-Harris and Farrar, a sample of 128 patients with ALL showed elevated levels of STAT5 phosphorylation, demonstrating that the protein plays a key role in the transformation of progenitor B cells. High levels of phosphorylated STAT5 were negatively correlated with survival. In addition, cooperation between STAT5 and mutated forms of EBF1 and PAX5 (important transcription factors for the development of B cells) was linked to the initiation of B cell transformation (1, 139).

Hyperactivation of the JAK2-STAT5 pathway can also lead to myeloproliferative neoplasms (MPNs)—a disease of hematopoietic stem cells (140). One of the most common mutations, V617F, in JAK2 has been directly linked to the constitutive activation of STAT5 (141). This mutated JAK2 requires the presence of thrombopoietin receptor (TPO-R) to promote the cytokine-independent activation of STAT5 and the MPN phenotype (141). Understanding the relationship between JAK2-STAT5 activation in MPNs can help expand treatment options for patients including ruxolitinib and other JAK inhibitors (142).

STAT5 also regulates apoptosis in cancer cells, and when it is constitutively active, mutant cells have properties of transformed cancer cells (143, 144). STAT5 activation has been shown to turn on *Bcl-xL*, a gene with an anti-apoptotic role, leading to the development of aggressive solid tumors (145). The link between activation of STAT5a and STAT5b to the progression of solid tumors in breast and prostate cancer, respectively, is clear but the mechanism behind the developments of these cancers requires further elucidation. The regulatory role of STAT5a during mammary gland remodeling and cell death has been well documented. Additionally, mouse models involving constitutively activated STAT5a further emphasize the transcription factor’s involvement in tumorigenesis (146). This constitutively active state of STAT5a has also been detected in humor breast cancer cell lines (147). STAT5b, on the other hand, is directly linked to the development and growth of prostate cancer. Along with being critical for the survival of prostate cancer cells, high levels of active STAT5b are also linked to early recurrence of the disease. Substantial cell death occurs in prostate cancer cells when the activation of STAT5b is inhibited (148–150). Being able to directly link this key transcription factor to the development of breast and prostate cancers has opened new doors for individualized treatment plans with an opening for the expanded landscape of biologic precision therapy. For this reason, it is critical to continue expanding our understanding of how the activation of STAT5 can promote tumorigenesis.

Evidence also suggests that STAT5 is involved in epithelial-mesenchymal transition (EMT), an important step in the process of tumor invasion. Because EMT plays such a significant role in the development of aggressive hepatocellular carcinoma (HCC), studies have been conducted linking STAT5b to the invasive properties of HCC (145). Along with solid breast and prostate tumors, dysregulation in the JAK/STAT5 pathway has been directly linked to the development of osteosarcoma (OS), a common primary bone tumor. In a study by Subramaniam and colleagues, they showed that treatment of cells with pimozide, a STAT5 inhibitor, lead to suppressed growth of OS cells. These results emphasized STAT5’s role in the development of OS and offer promising treatment options (151).

Continued research into the mechanism by which STAT5 activation contributes to the development of cancers and a better understanding of the downstream effects of dysregulated STAT5 activation may lead to the development of more accurate and effective therapeutic targets.

## Conclusions

STAT5b is a master regulator in the hemopoietic compartment as well as an essential signaling partner in extra-hematopoietic tissue regulation and growth. The critical role of STAT5 in lymphocyte development, GM-CSF signaling, and growth highlights the importance of studying these mechanisms in correlation with human diseases. Enhanced knowledge on STAT5 activation and regulation will guide genomic sequencing, improve early diagnosis and targeted therapeutic interventions.

## Author contributions

All authors listed have made a substantial, direct, and intellectual contribution to the work, and approved it for publication.
